# The relationship between HIV and fertility in the era of antiretroviral therapy in sub‐Saharan Africa: evidence from 49 Demographic and Health Surveys

**DOI:** 10.1111/tmi.12983

**Published:** 2017-10-24

**Authors:** M. Marston, B. Zaba, J. W. Eaton

**Affiliations:** ^1^ London School of Hygiene and Tropical Medicine London UK; ^2^ Department of Infectious Disease Epidemiology Imperial College London UK

**Keywords:** fertility, HIV, demographic and Health surveys, HIV Infections/prevention and control, antiretroviral therapy, highly active, HIV infections/therapy, fertilité, VIH, enquêtes démographiques et de santé, infections VIH/prévention et lutte, thérapie antirétrovirale, très efficace, infections VIH/thérapie

## Abstract

**Objectives:**

To describe regional differences in the relative fertility of HIV‐positive *vs*. HIV‐negative women and changes as antiretroviral treatment (ART) is scaled up, to improve estimates of predicted need for and coverage of prevention of mother‐to‐child transmission services at national and subnational levels.

**Methods:**

We analysed 49 nationally representative household surveys in sub‐Saharan Africa between 2003 and 2016 to estimate fertility rate ratios of HIV‐positive and HIV‐negative women by age using exponential regression and test for regional and urban/rural differences. We estimated the association between national ART coverage and the relationship between HIV and fertility.

**Results:**

Significant regional differences exist in HIV and fertility relationships, with less HIV‐associated subfertility in Southern Africa. Age patterns of relative fertility are similar. HIV impact on fertility is weaker in urban than rural areas. For women below age 30, regional and urban/rural differences are largely explained by differences in age at sexual debut. Higher levels of national ART coverage were associated with slight attenuation of the relationship between HIV and fertility.

**Conclusions:**

Regional differences in HIV‐associated subfertility and urban–rural differences in age patterns of relative fertility should be accounted for when predicting need for and coverage of PMTCT services at national and subnational level. Although HIV impacts on fertility are somewhat reduced at higher levels of national ART coverage, differences in fertility between HIV positive and negative remain, and fertility of women on ART should not be assumed to be the same as HIV‐negative women. There were few data in recent years, when ART has reached high levels, and this relationship should continue to be assessed as further evidence becomes available.

## Abstract (fr)

**Objectifs:**

Décrire les différences régionales dans la fécondité relative des femmes VIH positives versus celles négatives pour le VIH et les changements alors que le traitement antirétroviral (ART) est déployé, afin d'améliorer les estimations des besoins prévus et de la couverture de la prévention des services de prévention de la transmission mère‐enfant (PTME) au niveau national et infranational.

**Méthodes:**

Nous avons analysé 49 enquêtes nationales représentatives auprès des ménages en Afrique subsaharienne entre 2003 et 2016 pour estimer les rapports de taux de fécondité des femmes VIH positives et négatives par âges en utilisant une régression exponentielle et un test pour les différences régionales et urbaines/rurales. Nous avons estimé l'association entre la couverture ART nationale et relation entre le VIH et la fertilité.

**Résultats:**

Des différences régionales importantes existent dans les relations entre le VIH et la fertilité, avec peu d'hypofertilité associée au VIH en Afrique australe. Les profils d’âge de la fécondité relative sont similaires. L'impact du VIH sur la fécondité est plus faible dans les zones urbaines que dans les zones rurales. Chez les femmes de moins de 30 ans, les différences régionales et urbaines/rurales s'expliquent en grande partie par les différences d’âge du début de la sexualité. Des niveaux plus élevés de la couverture ART nationale ont été associés avec une légère atténuation de la relation entre le VIH et la fertilité.

**Conclusions:**

Les différences régionales dans l’ hypofertilité associée au VIH et les différences urbaines/rurales dans les profils d’âge de la fécondité relative devraient être prises en compte lors de la prévision des besoins et de la couverture des services de PTME au niveau national et infranational. Bien que les impacts du VIH sur la fécondité soient quelque peu réduits dans des niveaux plus élevés de couverture ART nationale, les différences de fécondité entre VIH positif et négatif persistent, et la fertilité des femmes sous ART ne devrait pas être considérée comme identique à celles des femmes VIH négatives. Il y avait peu de données au cours des dernières années lorsque l’ART a atteint des niveaux élevés et l’évaluation de cette relation doit continuer maintenant que de nouvelles données deviennent disponibles.

## Introduction

Elimination of mother‐to‐child transmission of HIV (MTCT) through provision of antiretroviral treatment (ART) to all HIV‐positive pregnant women is a major policy objective for national HIV programmes: The Joint United Nations Programme on HIV/AIDS (UNAIDS) and the US President's Emergency Plan for AIDS Relief (PEPFAR) 1. Accurate estimates of the number of HIV‐positive pregnant women at the national and subnational level are essential for planning and allocating resources needed for services to prevent mother‐to‐child transmission (PMTCT), calculating coverage and unmet need of existing services and evaluating progress towards elimination targets 2. Estimates of PMTCT need, coverage and MTCT rates are key outputs of official annual national HIV programme reports, generated with support from UNAIDS 3. HIV prevalence in antenatal care and PMTCT settings is the main indicator of national HIV epidemic trends, but in order to interpret it correctly as a guide to prevalence in the general population, we need to understand the relative incidence of pregnancy in HIV‐positive and HIV‐negative women.

Coverage and unmet need for PMTCT services are estimated by dividing number of pregnant women receiving PMTCT services from routine programmatic data (the numerator) by a modelled estimate of the number of HIV‐positive pregnant women (the denominator). Estimating the number of HIV‐positive pregnant women, and hence need for PMTCT services, relies on information about (i) age‐ and sex‐specific HIV prevalence in the population, (ii) age‐specific fertility rates and (iii) the fertility of HIV‐positive women relative to HIV‐negative women. Existing literature about the effects of HIV status on fertility emphasises a changing relationship with age 4, 5, 6, 7, 8. At the youngest ages, HIV‐positive women, relative to HIV‐negative women, have higher fertility due to selection of sexually active women. The fertility of HIV‐positive women relative to HIV‐negative women steadily declines with age, due to both biological effects of HIV on fecundity 8, 9, 10 and differences in exposure to pregnancy including factors such as higher divorce rates and widowhood in HIV‐positive women 11. A few studies have suggested regional differences in HIV‐related subfertility 7, 12, 13, although regional variation, is not systematically accounted for in current estimates of PMTCT need.

It is widely anticipated that ART scale‐up will ameliorate the subfertility of HIV‐positive women, which would affect the number of HIV‐positive pregnant women, although evidence of this is limited 14. In the era of ART, most studies of the impact of ART on pregnancy or fertility have been clinic‐based 14 which have shown some evidence that fertility increases after the first year on ART but still remains lower than in HIV‐negative women. Elul *et al*. 15 have criticised existing evidence from clinical cohorts that do not account for the effect of pregnancy status at enrolment. Allowing for this in an analysis of 26 clinics in East Africa, they found little evidence that ART initiation is associated with an increased risk of pregnancy in women who enrol in HIV care. A number of population‐level studies have shown evidence of a narrowing of fertility differences between HIV‐positive and HIV‐negative women 13, 16 in the era of ART.

This study aimed to improve the characterisation of the relative fertility of HIV‐positive women to HIV‐negative women by region and place of residence and update widely used estimates with data from the ART era.

## Methods

### Data

We used data from 49 Demographic and Health Surveys (DHS) and AIDS indicator surveys (AIS) conducted in 27 sub‐Saharan African countries between 2003 and 2016 in which both full birth histories and HIV testing outcomes were available 17. National ART coverage estimates for adult women were taken from UNAIDS estimates 18 and ranged from none in the earlier years to 72% in Zimbabwe in 2015 (Table 1).

**Table 1:** Demographic and health surveys with HIV women testing population samples by September 2017

Region	Survey	Year	*n*	HIV prevalence women 15–49 (95% CI)^a^	Estimated female adults 15+ ART coverage^b^ (%)^b^(18)	Median age at first sex 25–29‐year‐olds^c^		
						Urban	Rural	All
Southern Africa	Lesotho	2004	3030	26.3 (24.5–28.2)	1 (1–1)	19.0	18.6	18.7
	Lesotho	2009	3778	26.7 (25.0–28.6)	27 (25–29)	18.9	18.3	18.5
	Lesotho	2014	3175	29.7 (27.7–31.8)	40 (37–43)]	18.8	18.3	18.5
	Namibia	2013	4051	16.9 (15.4–18.4)	62 (50–70)	19.0	18.3	18.8
	Swaziland	2006–07	4424	31.1 (29.4–32.9)	10 (8–11)	18.6	17.9	18.1
	Zimbabwe	2005–06	6947	21.1 (19.7–22.6)	2 (2–3)	19.7	18.4	18.9
	Zimbabwe	2010–11	7313	17.7 (16.6–18.8)	31 (24–38)	20.5	18.6	19.3
	Zimbabwe	2015	8667	16.7 (15.6–17.8)	72 (57–84)	20.0	17.9	18.6
East and mid‐Africa	Burundi	2010	4533	1.7 (1.4–2.1)	33 (26–40)	20.7	19.9	19.9
	Kenya	2003	3151	8.7 (7.6–10.0)	0 (0–0)	18.9	17.6	18.0
	Kenya	2008–09	3641	8.0 (6.8–9.3)	16 (15–18)	19.5	17.7	18.3
	Malawi	2004	2686	13.3 (12.0–14.8)	1 (1–2)	18.2	17.4	17.5
	Malawi	2010	7091	12.9 (11.8–14.1)	31 (29–33)	17.9	17.1	17.3
	Malawi	2015–16	7737	10.8 (9.9–11.7)	66 (63–71)	18.1	16.9	17.2
	Rwanda	2005	5641	3.6 (3.1–4.2)	9 (8–11)	20.3	19.9	20.0
	Rwanda	2010	6917	3.7 (3.3–4.2)	45 (39–51)	21.5	21.3	21.3
	Rwanda	2014–15	6752	3.6 (3.2–4.1)	67 (59–76)	21.4	21.5	21.5
	Tanzania	2007–08	8179	6.6 (5.9–7.4)	10 (8–12)	18.2	17.3	17.5
	Tanzania	2011–12	9756	6.2 (5.6–6.8)	24 (18–28)	18.3	17.3	17.9
	Zambia	2007	5502	16.1 (14.7–17.5)	20 (19–22)	17.9	17.0	17.4
	Zambia	2013–14	14719	15.1 (14.2–16.0)	53 (50–56)]	18.3	16.9	17.5
West and central Africa and Ethiopia	Burkina	2003	4086	1.5 (1.2–2.0)	1 (1–1)	18.4	17.3	17.4
	Burkina	2010	8298	1.2 (0.9–1.5)	32 (25–40)	18.6	17.3	17.6
	Cameroon	2004	5128	6.6 (5.9–7.4)	2 (2–3)	17.1	15.8	16.5
	Cameroon	2011	7221	5.6 (5.0–6.3)	18 (15–20)	17.7	16.5	17.3
	Chad	2014–15	5656	1.8 (1.4–2.2)	50 (42–59)	16.7	16.1	16.2
	Cote Ivoire	2005	4413	6.4 (5.5–7.5)	6 (5–7)	16.9	16.1	16.4
	Cote Ivoire	2011–12	4509	4.6 (3.9–5.4)	25 (22–27)	17.6	16.3	16.9
	DRC	2007	4492	1.6 (1.2–2.2)	5 (4–6)	17.4	16.4	16.9
	DRC	2013–14	9264	1.6 (1.2–2.2)	24 (19–29)	17.4	16.4	16.8
	Ethiopia	2005	5736	1.9 (1.4–2.4)	2 (2–3)	20.7	16.1	16.6
	Ethiopia	2011	14695	1.9 (1.5–2.3)	41 (32–51)	18.3	17.2	17.4
	Gabon	2012	5459	5.8 (4.7–7.1)	32 (26–38)	17.1	16.7	17.1
	Gambia	2013	4089	2.1 (1.6–2.8)	24 (18–30)	20.6	18.0	19.3
	Ghana	2003	5097	2.3 (1.9–2.8)	0 (0–0)	19.4	17.9	18.3
	Guinea	2005	3742	1.9 (1.4–2.6)	2 (1–2)	16.7	15.9	16.0
	Guinea	2012	4622	2.1 (1.7–2.6)	28 (21–34)	17.6	15.7	16.3
	Liberia	2007	6382	1.8 (1.4–2.1)	3 (2–3)	16.6	16.1	16.3
	Liberia	2013	4397	2.0 (1.5–2.8)	19 (15–24)	16.6	16.0	16.4
	Mali	2006	4528	1.4 (1.0–2.0)	8 (6–10)	16.8	15.9	16.2
	Mali	2012–13	4806	1.3 (1.0–1.8)	32 (24–40)]	18.0	16.5	16.8
	Niger	2006	4406	0.6 (0.4–0.9)	3 (2–4)	17.9	15.6	15.8
	Niger	2012	5000	0.4 (0.2–0.5)	27 (20–32)	18.7	15.8	16.0
	Sao Tome	2009	2378	1.3 (0.8–2.0)		17.6	17.3	17.5
	Senegal	2005	4229	0.7 (0.4–1.0)	0 (0–0)	21.2	17.5	19.3
	Senegal	2010–11	5326	0.6 (0.4–0.8)	33 (25–40)	21.1	17.9	19.4
	Sierra Leone	2008	3448	1.7 (1.3–2.3)	4 (3–5)	16.7	15.7	16.0
	Sierra Leone	2013	7695	1.7 (1.3–2.0)	21 (13–29)	17.0	16.0	16.4
	Togo	2013–14	4737	3.1 (2.6–3.7)	37 (27–49)	18.6	17.4	18.1

a Estimated HIV prevalence, see Methods section.

b http://aidsinfo.unaids/, accessed 07 September 2017. Note for those surveys running over two years the earlier year is given.

c ICF International, 2015. The DHS Program STATcompiler. http://www.statcompiler.com. September 07 2017.

DHS and AIS are nationally representative household surveys 17. All analyses account for the two‐stage cluster sampling survey design and use the HIV weights provided by DHS. In pooled analysis, surveys are reweighted so that each survey contributes equally towards the analysis.

### Calculating age‐specific fertility rates

Each woman respondent was asked birth history questions for up to 20 births, beginning with the most recent. Dates of birth of the women and children are given in months and years, we assigned the day of birth to be the mid‐point of the month.

We initially analysed fertility rates by HIV status during the three years prior to the interview. This cut‐off was used in previous studies 4, 19 to balance the benefits of maximising the person years of observation while seeking to minimise maternal survivorship bias, recall bias and misclassification of HIV status over the three preceding years 7. However, we report results adjusted for the first year prior to the survey due to evidence of persistence of these biases when using data from longer than a year prior to the survey (see document, Supplemental Digital Content S1, which shows the analysis).

We used the standard demographic definition of age‐specific fertility rates (ASFR):

$${\mathrm{ASFR}}_{x-x+4}=\frac{\text{Number of births to women aged}\;x\;\mathrm{to}\;x+4}{\text{Number of person years contributed by women aged}\;x\;\mathrm{to}\;x+4}.$$

We then estimated the fertility rate ratios in the general population. Subsequently, we restricted the analysis to person years after first sex to assess the extent to which variation in age at first sex explains fertility differences among HIV‐positive women and HIV‐negative women in the younger age groups. We assumed sexual debut occurred on the date corresponding to the mid‐point of the reported age at first sex (which is reported as an integer age). Age at first sex was changed to nine months before the reported date of first birth if this was earlier than the mid‐point of reported age at first sex.

### Other variables

Other variables included women's HIV status at the time of the survey, calendar year, ART coverage, region and place of residence (urban/rural). Those infected with HIV‐2 and those whose HIV test was indeterminate were excluded from the analysis (0.04%).

Countries were grouped into regions as follows: Southern (Zimbabwe, Lesotho, Swaziland and Namibia), East and mid‐Africa (Tanzania, Kenya, Uganda, Rwanda, Burundi, Malawi and Zambia) and West and central Africa with Ethiopia (Table 1). HIV epidemics in the East and mid‐African countries occurred earlier than in Southern Africa. West and central Africa along with Ethiopia have lower prevalence, and HIV transmission is likely more concentrated.

ART coverage estimates, taken from UNAIDS estimates 20 of national female adult ART coverage at the time of each survey were stratified into categories <20%, 20–49% and >50% (Table 1). For surveys that ran over two different years, a mid‐point of the estimated coverage in both years was taken.

### Analysis

We used exponential regression to investigate the interaction between HIV status and five‐year age group, place of residence, region and ART coverage with respect to their impacts on fertility. Each analysis was adjusted for country and survey year. The analysis was repeated excluding person time prior to first sex. The multivariate Wald test was used to assess significance of interaction terms. The first model includes only the interaction between age and HIV status controlled for country and year of survey. Subsequent models include the effect of place of residence, region and national ART coverage and the interactions between them and age and HIV status.

All models included the three‐way interaction between year before the survey, five‐year age group and HIV status. Results are reported for the first year before the interview date. Analyses were conducted using Stata version 14.1

## Results

### Pooled analysis of DHS surveys

Model 1 estimated the crude fertility rate ratio (FRR) for HIV‐positive women relative to HIV‐negative women by five‐year age group across all countries and surveys, adjusted only for calendar year and country. In the 15‐ to 19‐year age group, fertility was 1.38 (95% CI 1.19–1.61) times higher in HIV‐positive women than HIV‐negative women, consistent with the fact that for younger ages many women have not yet been exposed to sex and therefore neither to HIV. Thereafter, fertility of HIV‐positive relative to HIV‐negative women decreased with age from an FRR of 0.93 (95% CI 0.85–1.03) in 20‐ to 24‐year‐olds to 0.29 (95% CI 0.13–0.66) in 45‐ to 49‐year‐olds (Supplemental Digital Content 2: Figure A2, show stratum‐specific ratios derived from Table 2, Model 1 along with estimates made by Chen and Walker 4).

**Table 2:** Adjusted fertility rate ratios for all women aged 15–49

	Model 1		Model 2	
	FRR	95% CI	FRR	95% CI
HIV status				
HIV negative	1		1	
HIV positive	0.70	0.63–0.78	0.62	0.53–0.73
Effects of HIV by age				
15–19, HIV positive	1.92	1.59–2.32	2.39	1.86–3.08
20–24, HIV positive	1.32	1.15–1.52	1.54	1.29–1.85
25–29, HIV positive	1.14	0.99–1.30	1.31	1.08–1.59
30–34, HIV positive	1		1	
35–39, HIV positive	0.76	0.63–0.92	0.80	0.62–1.04
40–44, HIV positive	0.62	0.43–0.87	0.67	0.41–1.09
45–49, HIV positive	0.41	0.18–0.95	0.10	0.01–0.94
Effects of HIV by place of residence				
Urban, HIV positive				1
Rural, HIV positive			1.18	1.02–1.36
Effects of place of residence on age and HIV status interaction				
Rural, HIV positive, 15–19			0.71	0.56–0.89
Rural, HIV positive, 20–24			0.77	0.65–0.92
Rural, HIV positive, 25–29			0.78	0.65–0.92
Rural, HIV positive, 30–34			1	
Rural, HIV positive, 35–39			0.91	0.71–1.16
Rural, HIV positive, 40–44			0.91	0.57–1.44
Rural, HIV positive, 45–49			4.71	0.60–36.8
Effects of HIV by region				
Southern, HIV positive			1.12	1.05–1.20
Eastern, HIV positive				1
Western, HIV positive			0.99	0.91–1.08
Effects of HIV by ART coverage				
<20%, HIV positive				1
20–49%, HIV positive			1.05	0.98–1.11
>50%, HIV positive			1.09	1.01–1.18
Age group				
15–19	0.57	0.55–0.60	0.50	0.47–0.53
20–24	1.08	1.04–1.12	0.98	0.93–1.03
25–29	1.13	1.09–1.17	1.11	1.05–1.16
30–34	1		1	
35–39	0.76	0.73–0.80	0.69	0.65–0.74
40–44	0.33	0.31–0.36	0.26	0.23–0.29
45–49	0.09	0.07–0.10	0.06	0.05–0.08
Place of residence				
Urban			1	
Rural			1.37	1.32–1.42
Effects of age by place of residence				
Rural, 15–19			1.25	1.18–1.32
Rural, 20–24			1.18	1.13–1.23
Rural, 25–29			1.04	1.00–1.09
Rural, 30–34			1	
Rural, 35–39			1.13	1.06–1.20
Rural, 40–44			1.36	1.21–1.53
Rural, 45–49			1.58	1.21–2.06
Region				
Southern			0.67	0.64–0.70
Eastern			1	
Western			0.72	0.68–0.77
ART coverage				
<20%			1	
20–49%			0.96	0.93–1.00
>50%			0.77	0.73–0.82

Results from exponential regression of fertility rates as a function of HIV status, age controlling for country and calendar year. Also not shown is the additional interaction between years before the survey, HIV status and age group. Model 2 has an additional interaction between place of residence, age group and HIV status, region and HIV status, and ART coverage and HIV status.

### Variation by region and place of residence

There was a significant interaction between HIV status and region (Table 2, Model 2). In Southern Africa, the relative fertility rate of HIV‐positive compared to HIV‐negative women was 1.12 (95% CI 1.05–1.20) times higher than in the Eastern region (Table 2, Model 2). West and central countries were similar to East and mid‐Africa (RR 0.99, 95% CI 0.91–1.08; Table 2, Model 2 and Figure 2). There was no significant interaction between region, five‐year age group and HIV status (Wald test *F* = 1.37, *P* = 0.173), indicating lack of evidence of regional difference in the relative age pattern of HIV subfertility.

**Figure 1:**
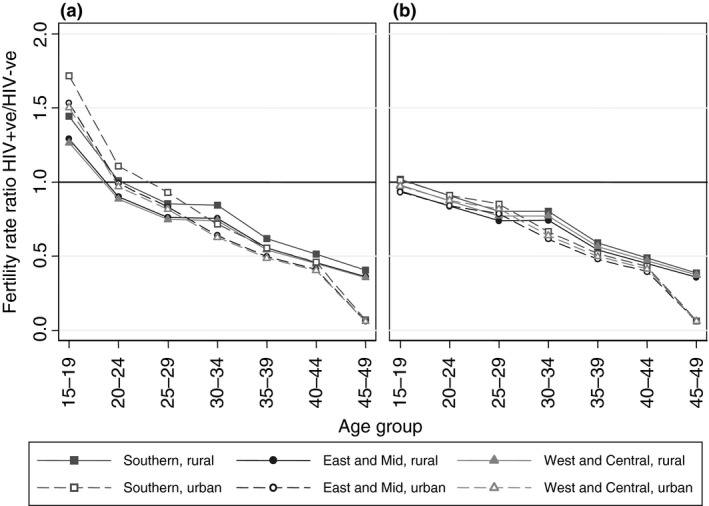
Adjusted age‐specific fertility rate ratios comparing HIV‐positive women to HIV‐negative women by region and urban and rural residence (adjusted for region, place of residence, the effect of five‐year age group on HIV status and place of residence, ART coverage, country, calendar year) using all women person years (a) and excluding person years prior to first sex (b).

For all surveys except two, fertility rates are higher in rural areas than urban areas for 15‐ to 49‐year‐old women, whilst HIV prevalence is lower in rural than to urban areas (see Supplemental Digital Content S2: Figure A1). The statistically significant effect of place of residence on the relationship between HIV and fertility (Table 2, Model 2) indicates that these systematic urban/rural differences in fertility and HIV partially explains the overall lower fertility of HIV‐positive women. In contrast to region, place of residence did significantly affect the age pattern of relative fertility (Wald test *F* = 2.80, *P* = 0.010), with a steeper gradient in urban areas than in rural areas (Figure 1a). Among 30‐ to 34‐year‐olds, the relative fertility of women in rural areas was 1.18 (95% CI 1.02–1.36) times greater than in urban areas, while among 15‐ to 19‐year‐olds relative fertility of HIV‐positive women was 0.83 (95% CI 0.69–0.995) times lower in rural areas than urban.

### ART coverage and HIV subfertility

**Figure 2:**
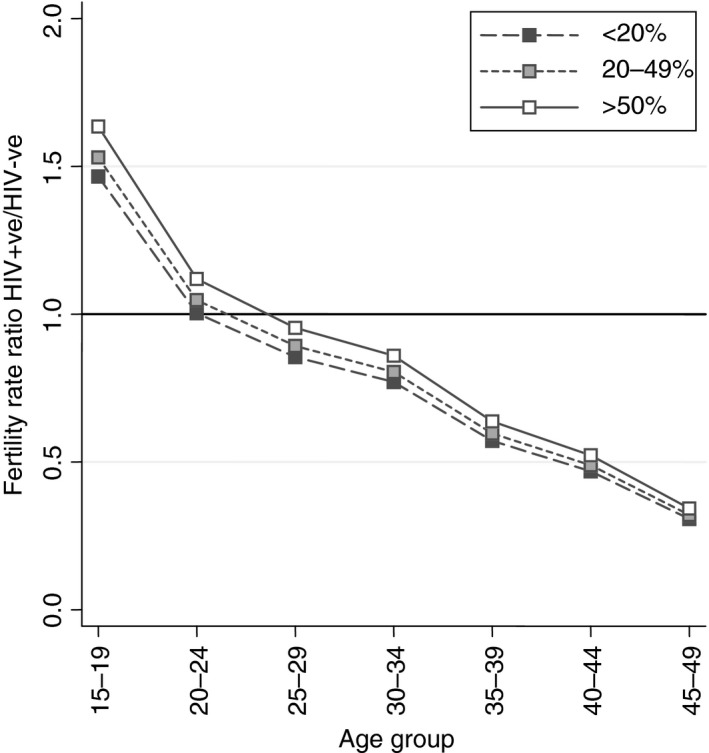
Adjusted age‐specific fertility rate ratios for Southern Africa comparing HIV‐positive women to HIV‐negative women by region and national ART coverage (adjusted for region, place of residence, the effect of five‐year age group on HIV status and place of residence, ART coverage, country, calendar year).

The fertility differences between HIV‐positive and HIV‐negative women slightly reduced as ART coverage increased. With ART coverage at over 50%, the fertility rate ratio was 1.09 times higher (95% CI 1.01–1.18), compared to when ART coverage was below 20%. However, overall the fertility of HIV‐positive women remained significantly lower than that of HIV‐negative women in recent surveys with high ART coverage. For example, in urban Southern Africa, the fertility rate ratio increased from 0.70 (95% CI 0.59–0.82) in 30‐ to 34‐year‐olds in a time with <20% national ART coverage to 0.76 (95% CI 0.64–0.91) when there was 50% ART coverage (Figure 2, Table 2; Supplemental Digital Content S2: Table A1). There was no evidence that the level of ART coverage affected the age pattern of relative fertility (Wald test *F* = 1.23, *P* = 0.253).

### HIV and fertility among women who have ever had sex

Across surveys, we observed consistently higher median age at first sex among young women in urban areas compared to rural areas and in southern African countries compared to east Africa and west and central Africa (Table 1). This suggests that some of the variation in the relative fertility of HIV‐positive women at the youngest ages (Figure 1a) may be attributable to systematic differences in the age at sexual debut. We replicated the above models, excluding person years prior to first sex (Table 3). Removing the person years of women who had not become sexually active completely explained the higher fertility among HIV‐positive women aged under 25 compared to HIV‐negative women (Figure 1b shows the stratum‐specific rate ratios derived from Table 3, Model 2). In all regional, place of residence and ART coverage groups, the fertility of HIV‐positive women aged 15–19 was not significantly different to that of HIV‐negative women when excluding person time prior to first sex (Table 3, Figure 1b; Supplemental Digital Content S2: Table A2). Excluding person time prior to first sex gave a relative fertility rate ratio of 0.92 (95% CI 0.76–1.11) for 15‐ to 19‐year‐old women in urban areas, compared to 1.49 (95% CI 1.22–1.82) when analysing all women. Similarly, in rural areas, the FRR for 15‐ to 19‐year‐olds was 0.89 (95% CI 0.76–1.05) after excluding person years prior to first sex, compared to 1.23 (95% CI 1.04–1.47) for all person years. For 20‐ to 24‐year‐olds, the relative fertility of HIV‐positive women fell, and in many cases, it was significantly lower than that of HIV‐negative women (*P* < 0.05) when restricted to sexually active women (Figure 2; Supplemental Digital Content S2: Table A2). The effect of age on the interaction between places of residence remained but was reduced at younger ages. After restricting to person years for women after first sex, the differences in the relative fertility for women under age 25 by place of residence and differences by region were substantially reduced (Figure 2; Supplemental Digital Content S2: Tables A1 and A2). For example, for 15‐ to 19‐year‐olds, the relative fertility rate ratio was the same in the urban and rural areas (interaction term 0.95, 95% CI 0.80–1.13 compared to 0.83, 95% CI 0.69–0.995 when including all women's person time).

**Table 3:** Adjusted fertility rate ratios for those women aged 15–49 excluding person years prior to first sex

	Model 1		Model 2	
	FRR	95% CI	FRR	95% CI
HIV status				
HIV negative	1		1	
HIV positive	0.69	0.62–0.77	0.59	0.51–0.69
Effects of HIV by age				
15–19, HIV positive	1.33	1.11–1.60	1.55	1.21–1.98
20–24, HIV positive	1.20	1.05–1.38	1.36	1.13–1.63
25–29, HIV positive	1.12	0.97–1.29	1.29	1.06–1.56
30–34, HIV positive	1		1	
35–39, HIV positive	0.77	0.63–0.92	0.81	0.62–1.04
40–44, HIV positive	0.62	0.44–0.88	0.67	0.41–1.10
45–49, HIV positive	0.42	0.18–0.96	0.10	0.01–0.96
Effects of HIV by place of residence				
Urban, HIV positive				1
Rural, HIV positive			1.21	1.05–1.40
Effects of place of residence on age and HIV status interaction				
Rural, HIV positive, 15–19			0.78	0.63–0.98
Rural, HIV positive, 20–24			0.82	0.69–0.98
Rural, HIV positive, 25–29			0.78	0.65–0.93
Rural, HIV positive, 30–34			1	
Rural, HIV positive, 35–39			0.90	0.71–1.16
Rural, HIV positive, 40–44			0.90	0.57–1.42
Rural, HIV positive, 45–49			4.65	0.6–36.32
Effects of HIV by region				
Southern, HIV positive			1.09	1.02–1.16
Eastern, HIV positive				1
Western, HIV positive			1.05	0.96–1.14
Effects of HIV by ART coverage				
<20%, HIV positive				1
20–49%, HIV positive			1.04	0.98–1.11
>50%, HIV positive			1.13	1.05–1.22
Age group				
15–19	1.20	1.15–1.24	1.16	1.10–1.22
20–24	1.22	1.17–1.26	1.16	1.10–1.22
25–29	1.15	1.11–1.20	1.14	1.08–1.20
30–34	1		1	
35–39	0.76	0.73–0.80	0.69	0.64–0.74
40–44	0.33	0.31–0.35	0.25	0.23–0.28
45–49	0.08	0.07–0.10	0.06	0.04–0.07
Place of residence				
Urban			1	
Rural			1.32	1.27–1.37
Effects of age by place of residence				
Rural, 15–19			1.05	1.00–1.10
Rural, 20–24			1.08	1.04–1.13
Rural, 25–29			1.03	0.98–1.08
Rural, 30–34			1	
Rural, 35–39			1.13	1.06–1.21
Rural, 40–44			1.37	1.22–1.53
Rural, 45–49			1.59	1.22–2.07
Region				
Southern			0.74	0.71–0.77
Eastern			1	
Western			0.72	0.68–0.76
ART coverage				
<20%			1	
20–49%			0.96	0.92–0.99
>50%			0.76	0.72–0.80

Results from exponential regression of fertility rates as a function of HIV status, age controlling for country and calendar year. Also not shown is the additional interaction between years before the survey, HIV status and age group. Model 2 has an additional interaction between place of residence, age group and HIV status, region and HIV status, and ART coverage and HIV status.

## Discussion

This analysis has shown that overall subfertility attributable to HIV is slightly less pronounced than previously thought, and we find that it varies across settings. Consistent regional and urban/rural differences have been found, which are largely explained by variation in age at first sex. The fertility differential between HIV‐positive and HIV‐negative women appears to have narrowed in recent years as ART coverage has increased; however, caution is required in attributing this directly to ART.

We corroborated patterns found in previous studies showing increasing HIV‐associated subfertility with age 4, 6, 7. Also consistent with these other studies, we find that in the youngest age group, HIV‐positive women have higher fertility than their HIV‐negative counterparts as many women in this age group are not sexually active and therefore are not exposed to either HIV or pregnancy. Chen and Walker reported a strong relationship between the percentage of 15‐ to 19‐year‐olds who are sexually active and the fertility rate ratio among this age group 4. We extended this to show that when restricting analysis of fertility to women who had sexually debuted, there was no difference in the fertility of HIV‐positive and HIV‐negative women, suggesting that selection for sexually active women completely explains the increased fertility of HIV‐positive women in this age group. Variation in age at first sex largely explained regional and place of residence differences in relative fertility. West and central Africa overall have a much lower median age at first sex than both East and Southern Africa at around 15–16 years *vs*. 18–19 years in the southern African countries (Table 1). The median age at sexual debut is older in urban than rural areas (Table 1), and again, once person years before sexual debut are removed from the analysis, there is no significant difference in relative fertility between urban and rural residency for women under 30. At older ages, HIV‐associated subfertility is more pronounced in the urban areas. This could be explained by differences in sexual activity between rural and urban areas 21, influenced by differences in social norms, desired family size, knowledge of HIV status and access to services that may influence contraceptive use or abstinence from sex. In addition to systematic differences in sexual debut, regions also differed in the scale of HIV epidemics and the stage of the epidemic at the time when surveys have been collected.

This analysis suggests that the relationship between HIV and fertility has attenuated slightly as the introduction of ART, but overall fertility remains significantly lower among HIV‐positive women than HIV‐negative women. These reductions are somewhat less dramatic than predicted by current estimates of PMTCT need published by UNAIDS using the Spectrum model, which assumes that women on ART for more than six months have the same fertility as HIV‐negative women of the same age. For example, under this assumption, a 50% increase in ART coverage would attenuate the overall FRR of HIV‐positive women from 0.7 times that of HIV‐negative women to 0.85. This is an increase of 1.21 times, somewhat greater than the 1.11 times increase that we estimated for survey periods with ART coverage >50% compared to those <20%. There was no evidence that the effect of ART coverage on HIV subfertility varied by age. As the differences in HIV‐associated subfertility by national ART coverage are small, it is possible that we did not have the power to detect any further differences by age. National ART coverage is an ecological variable. It does not measure individual exposure to treatment, and hence, we are cautious about attributing causality, for example countries with better roll out of ART may also have other things in common such as good health systems, with better provision of family planning services.

We find substantially less HIV‐associated subfertility than Chen and Walker 4 in women aged below 35 and more HIV‐associated subfertility at older ages. A number of factors can explain these differences. Chen and Walker had fewer surveys than were used in this analysis and did not adjust for place of residence or region, which we showed confounds the relationship between HIV and fertility because of systematically lower fertility in urban areas which also have higher HIV prevalence. The surveys used in the Chen and Walker analysis were predominantly from East, West and central Africa, where HIV‐associated subfertility is more pronounced than in Southern Africa. We looked at data for the three years prior to the survey when constructing our models as did Chen and Walker; however, we only report results from the first year before the survey due to evidence that using data beyond one year exaggerated the HIV‐associated subfertility in younger women (see document, Supplemental Digital Content S1). We also find substantially lower subfertility using the DHS data than we did using data from the demographic surveillance sites in Eastern and Southern Africa 7. Much of these DSS data are from rural populations around Lake Victoria in East Africa that experienced early and severe HIV epidemics, all factors that we expect to be associated with greater subfertility based on this multicountry analysis.

A number of recommendations arise from these analyses for improving estimates and predictions of need for PMTCT services. We found evidence for variation across regions, with less HIV‐associated subfertility in Southern Africa, but no evidence of differences by age pattern. This suggests that scaling the estimated age pattern of relative fertility of HIV‐positive women to reflect overall prevalence among pregnant women observed through routine HIV testing may be a reasonable approach to calibrating and reflecting variation across countries and settings. There are significant differences in the pattern of relative fertility by urban/rural residency, which appeared to be largely explained by older sexual debut in urban areas. This should be accounted for in planning and allocating resources for PMTCT at the subnational level and evaluating local progress towards MTCT elimination. Finally, the relationship between HIV and fertility has attenuated slightly as ART has been introduced, supporting the current practice to account for ART coverage when predicting fertility of HIV‐positive women and need for PMTCT. However, overall the reductions are somewhat less dramatic than predicted by current Spectrum assumptions, and fertility remains lower among HIV‐positive women than HIV‐negative women at older ages. Overall, we have characterised the fertility patterns of HIV‐positive women over time and across regions in sub‐Saharan Africa as ART scaled up from the mid‐2000s through 2015. However, they could continue to evolve rapidly as HIV treatment and prevention programmes enter a new era. Improving timely data about the fertility patterns of HIV‐positive women and deeper understanding of the mechanisms underlying changes will be important to plan and evaluate PMTCT policy and monitor epidemic trends.

## Supporting information

Supplementary Digital Content 1.

Supplementary Digital Content 2.
