# Supplementary material for: The relationship between HIV and fertility in the era of antiretroviral therapy in sub‐Saharan Africa: evidence from 49 Demographic and Health Surveys
Source: Trop Med Int Health. 2017 Oct 24;22(12):1542–50. doi: 10.1111/tmi.12983 (PMC5716842; doi:10.1111/tmi.12983)
Supplement: Supplementary file 1 — Supplementary Digital Content 1. [file TMI-22-1542-s001.docx]

# **Supplementary Digital Content 1:**

#

**“The relationship between HIV and fertility in the era of antiretroviral therapy in sub Saharan Africa – Evidence from 48 Demographic & Health Surveys”**

**Milly Marston, Basia Zaba, Jeffrey W. Eaton**

# **Possible biases in the analysis of HIV and fertility**

**Introduction**

Analysis of population level effects of HIV on fertility has relied on two types of studies, first retrospective data from demographic and health surveys (DHS)^1^ with HIV testing^2^, second data from community based HIV cohort studies^3, 4^. Demographic and Health Surveys have the advantage that they are large nationally representative surveys across sub Saharan Africa whereas community based cohort studies are usually not nationally representative and do not cover much of sub-Saharan Africa. However, community based HIV cohort studies could be seen as the gold standard as they prospectively test participants for HIV and record births and other demographic characteristics. This means that HIV status of the mother at the birth of the child is known, in contrast to using retrospective data, which requires us to assume that the HIV status of the women at time of interview is the same as her HIV status in the time prior to the survey. Retrospective surveys are also subject to survivorship bias, since not all women of childbearing age in the sample households survive to time of interview. Women who die prematurely are likely to have experienced a serious illness, such as AIDS, and during the time of serious illness they are likely to have low fertility. Both data sources can be affected by underreporting of young infant deaths, which are more likely to occur to HIV positive women, due to recall bias ^5^. However if we use only the most recent years before the surveys it is possible that DHS data are less affected by this bias as they directly ask the woman herself about her births as opposed to community based cohort studies which often use proxy respondents to report experiences of family members.

**Methods**

*Identifying biases in retrospective data used to analyse HIV subfertility*

We started the analysis reported in the manuscript using the standard cut off of three years prior to the survey. This standard is used by DHS in reporting general fertility rates^6^, and was adopted by previous studies of HIV and fertility^2^. This cut off for fertility analysis is chosen to balance the desire for maximizing the retrospective person years of observation while minimizing biases from using retrospective data. Table A1 summarises the potential biases that are known to occur in using retrospective birth reports to analyse fertility, many of which vary with age.

Using the same methods as outlined in the main paper we used exponential regression to estimate the interaction between single years before the survey and its effect on the age pattern of HIV subfertility. The model included HIV status and five year age group, single years before the survey and adjusted for country and survey year.

*Table A1: Biases affecting the use of retrospective data to measure HIV-associated subfertility*

| **Nature of bias** | **Direction of bias** | **Age groups affected** |
| --- | --- | --- |
| Survivorship bias: women who have been infected for longer are more likely to have died before being interviewed. They are also more likely to have much lower fertility due to illness, but are not included in the analysis | Fertility of HIV positive women is over-estimated, so HIV-associated sub-fertility is understated | More in older women as they are more likely to have been infected for longer and are thus at higher risk of dying |
| Age eligibility: survey eligibility is limited to ages of 15-49, therefore women aged 47+ in the three years prior to the survey may no longer be eligible to participate on the survey date. The composition of the oldest age group skews to the younger ages, where fertility is higher. | Fertility of HIV positive women is over-estimated, so HIV-associated sub-fertility is understated | Only the 45-49 year old age group is affected |
| HIV status miss-classification (i): women who sero-convert in the analysis interval, and who have a birth before sero-conversion will be wrongly classified as contributing births (and person-years) to HIV positive fertility | Fertility of HIV positive women is over-estimated, so HIV-associated sub-fertility is understated | This bias would be greatest at ages in which HIV incidence is highest, generally ages 20-34. |
| HIV status miss-classification (ii): women who sero-convert in the analysis interval, and who have a birth after sero-conversion will have too many person-years (but not too few births) classified as contributing to HIV positive fertility | Fertility of HIV positive women is under-estimated, so HIV-associated sub-fertility is exaggerated | This bias will be greatest if sero-conversion occurs close to the age of sexual debut, or formation of first regular sexual union, when births are more likely to occur after sero-conversion, so the age group most strongly affected will be 15-24 |
| HIV status miss-classification (iii): Women who sero-convert in the analysis interval, and who have no birth will have too many person-years classified as contributing to HIV positive fertility. | Fertility of HIV positive women is under-estimated, so HIV-associated sub-fertility is exaggerated | This bias would be greatest at ages in which HIV incidence is highest, generally ages 20-34. |
| Under-reporting of births of infants who die: Births that result in early neonatal and infant deaths tend to be underreported especially those which occurred further back in time. Since children of HIV positive women have higher mortality, especially before PMTCT services were widespread, this kind of under-reporting will be more frequent in HIV positive women | Fertility of HIV positive women is under-estimated, so HIV-associated sub-fertility is exaggerated | Affects all age groups, but likely to diminish in importance over time, as roll-out of PMTCT services improves mortality of children of HIV positive mothers |

Results

*Analysis of biases using retrospective data to analyse HIV subfertility*

First, we investigated possible biases in using the retrospective data for three years prior to the survey. We fitted a simple model with HIV status, age group and the interaction between the two, adjusted for country and calendar year as shown in the main paper (Table 2, Model 1). We introduced the variable representing the first, second and third year before the survey and tested it’s interaction with HIV status, this showed a significant decrease in the fertility rate ratio comparing HIV positive women to HIV negative women of 0.91 (0.85-0.98) and 0.94 (0.87-1.01) times in the second and third year compared to the first year respectively (Wald test for interaction p=0.073).

We also looked at how age affected the interaction between years before the survey and HIV status (Table A2, model 1). We found that the interaction between HIV status and years before the survey appeared to work in different directions. For women under 30 years HIV-associated subfertility was more pronounced if we used data further back than one year compared to the first year. The fertility rate ratio comparing positives to negatives decreasing by 0.86 (95%CI 0.79-0.94) and 0.89 (95%CI 0.81-0.97) in the 2^nd^ and 3^rd^ year respectively (Table A2, Model 2). For those aged 30 years and over, HIV-associated subfertility was less pronounced as a result of using data going back further than one year, although this was not significant (Table A2, Model 3). Table A3 and Figure A1 show the resultant adjusted age specific rate ratios by years used prior to the survey.

Discussion

We found evidence of biases when using retrospective data for analysis of subfertility. Using data beyond one year increased the subfertility in younger women and slightly increased it for older women although this was not significant. Older women are likely to have been infected with HIV for longer than younger women – this means that survivorship bias would be greater for older women, as with longer duration of infection they are likely to be less fertile ^4^ and less likely to survive to be interviewed. For younger women the assumption that they have been HIV positive for several years before the survey will be less true than for older women who are more likely to have sero converted long ago. Therefore the assumption of constant retrospective HIV status at younger ages will cause a larger misclassification of person years by HIV status: more negative person years will be wrongly classified as HIV positive in younger women, which would tend to decrease the apparent extent of their subfertility. However, we found the overall bias in the measurement of subfertility in younger women related to increasing the number of years of retrospective data used in the analysis went in the opposite direction. One possible reason for this is that the HIV negative time for younger women immediately prior to sero conversion is dominated by time prior to entry into first sexual union, or indeed prior to sexual debut, when women are not yet exposed to risk of conception or HIV acquisition. This coupled with all the person years when a women does not have a birth during a sero conversion interval being assigned to HIV positive women, would tend to exaggerate the extent of subfertility in the HIV positive group mirroring our observations.

Researchers should be aware of the many possible biases when analysing population based data on HIV and fertility, and try to minimise them. The biases found in this study show that biases in estimates of HIV sub fertility are strongly influenced by the age of the woman, and can be minimised by curtailing the analysis to the year immediately preceding the survey.

Table A2: Adjusted fertility rate ratios for all women, women under 30 and women over 30 to demonstrate the significant decrease in HIV subfertility in women under 30 when looking beyond one year prior to the survey.

|  | | All women | | |  | All women | | |  | Women <30 | | |  | Women 30+ | | |
| --- | --- | --- | --- | --- | --- | --- | --- | --- | --- | --- | --- | --- | --- | --- | --- | --- |
|  |  | FRR | 95 %CI | Wald |  | FRR | 95 %CI | Wald |  | FRR | 95 %CI | Wald |  | FRR | 95 %CI | Wald |
| *HIV status* |  |  |  |  |  |  |  |  |  |  |  |  |  |  |  |  |
|  | HIV negative |  |  |  |  | 1 |  |  |  | 1 |  |  |  | 1 |  |  |
|  | HIV Positive | 0.74 | (0.68-0.79) | *>0.001* |  | 0.70 | (0.63-0.78) | *>0.001* |  | 0.77 | (0.71-0.82) | *>0.001* |  | 0.73 | (0.67-0.80) | *>0.001* |
| *Age group* |  |  |  |  |  |  |  |  |  |  |  |  |  |  |  |  |
|  | 15-19 | 0.59 | (0.57-0.60) | |  | 0.57 | (0.55-0.59) | |  | 0.51 | (0.50-0.52) |  |  |  |  |  |
|  | 20-24 | 1.12 | (1.10-1.14) | |  | 1.08 | (1.04-1.12) | |  | 0.97 | (0.95-0.99) |  |  |  |  |  |
|  | 25-29 | 1.15 | (1.13-1.17) | |  | 1.13 | (1.09-1.17) | |  | 1.00 |  | *>0.001* |  |  |  |  |
|  | 30-34 | 1 |  |  |  | 1 |  |  |  |  |  |  |  | 1 |  |  |
|  | 35-39 | 0.76 | (0.75-0.78) | |  | 0.77 | (0.73-0.80) | |  |  |  |  |  | 0.76 | (0.75-0.78) |  |
|  | 40-44 | 0.35 | (0.34-0.36) | |  | 0.33 | (0.31-0.36) | |  |  |  |  |  | 0.35 | (0.34-0.36) |  |
|  | 45-49 | 0.11 | (0.10-0.12) | *>0.001* |  | 0.09 | (0.07-0.10) | *>0.001* |  |  |  |  |  | 0.11 | (0.10-0.12) | *>0.001* |
| *Effects of HIV by age* | |  |  |  |  |  |  |  |  |  |  |  |  |  |  |  |
|  | 15-19, HIV positive | 1.83 | (1.66-2.03) | |  | 1.97 | (1.64-2.37) | |  | 1.79 | (1.62-1.97) |  |  |  |  |  |
|  | 20-24, HIV positive | 1.22 | (1.12-1.32) | |  | 1.33 | (1.16-1.53) | |  | 1.18 | (1.09-1.27) |  |  |  |  |  |
|  | 25-29, HIV positive | 1.03 | (0.95-1.11) | |  | 1.13 | (0.98-1.29) | |  | 1 |  | *>0.001* |  |  |  |  |
|  | 30-34, HIV positive | 1 |  |  |  | 1 |  |  |  |  |  |  |  | 1 |  |  |
|  | 35-39, HIV positive | 0.81 | (0.73-0.91) | |  | 0.75 | (0.62-0.91) | |  |  |  |  |  | 0.81 | (0.73-0.91) |  |
|  | 40-44, HIV positive | 0.65 | (0.53-0.78) | |  | 0.60 | (0.43-0.85) | |  |  |  |  |  | 0.65 | (0.53-0.78) |  |
|  | 45-49, HIV positive | 0.41 | (0.24-0.69) | *>0.001* |  | 0.41 | (0.18-0.94) | *>0.001* |  |  |  |  |  | 0.41 | (0.25-0.69) | *>0.001* |
| *Year before survey* | |  |  |  |  |  |  |  |  |  |  |  |  |  |  |  |
|  | 1st | 1 |  |  |  | 1 |  |  |  | 1 |  |  |  | 1 |  |  |
|  | 2nd | 0.94 | (0.92-0.96) | |  | 0.89 | (0.86-0.94) | |  | 0.96 | (0.94-0.98) |  |  | 0.90 | (0.87-0.93) |  |
|  | 3rd | 0.95 | (0.94-0.97) | *>0.001* |  | 0.94 | (0.90-0.98) | *>0.001* |  | 0.96 | (0.94-0.98) | *>0.001* |  | 0.95 | (0.91-0.98) | *>0.001* |
| *Effects of HIV by years before survey* | |  |  |  |  |  |  |  |  |  |  |  |  |  |  |  |
|  | 2nd year, HIV positive | 0.92 | (0.86-0.99) | |  | 0.96 | (0.81-1.15) | |  | 0.87 | (0.79-0.95) |  |  | 1.03 | (0.91-1.18) |  |
|  | 3rd year, HIV positive | 0.94 | (0.88-1.01) | *0.073* |  | 1.05 | (0.90-1.21) | *0.639* |  | 0.89 | (0.82-0.97) | *0.003* |  | 1.04 | (0.93-1.17) | *0.764* |
| *Effects of yearbefore on age and HIV status interaction* | | | | |  |  |  |  |  |  |  |  |  |  |  |  |
|  | 2nd year, HIV positive, 15-19 | | |  |  | 0.94 | (0.71-1.24) | |  |  |  |  |  |  |  |  |
|  | 2nd year, HIV positive, 20-24 | | |  |  | 0.90 | (0.72-1.13) | |  |  |  |  |  |  |  |  |
|  | 2nd year, HIV positive, 25-29 | | |  |  | 0.89 | (0.71-1.11) | |  |  |  |  |  |  |  |  |
|  | 2nd year, HIV positive, 30-34 | | |  |  | 1 |  |  |  |  |  |  |  |  |  |  |
|  | 2nd year, HIV positive, 35-39 | | |  |  | 1.29 | (0.96-1.73) | |  |  |  |  |  |  |  |  |
|  | 2nd year, HIV positive, 40-44 | | |  |  | 1.03 | (0.63-1.69) | |  |  |  |  |  |  |  |  |
|  | 2nd year, HIV positive, 45-49 | | |  |  | 1.66 | (0.54-5.06) | |  |  |  |  |  |  |  |  |
|  | 3rd year, HIV positive, 15-19 | | |  |  | 0.86 | (0.67-1.11) | |  |  |  |  |  |  |  |  |
|  | 3rd year, HIV positive, 20-24 | | |  |  | 0.85 | (0.70-1.03) | |  |  |  |  |  |  |  |  |
|  | 3rd year, HIV positive, 25-29 | | |  |  | 0.85 | (0.70-1.05) | |  |  |  |  |  |  |  |  |
|  | 3rd year, HIV positive,30-34 | | |  |  | 1 |  |  |  |  |  |  |  |  |  |  |
|  | 3rd year, HIV positive, 35-39 | | |  |  | 0.99 | (0.76-1.29) | |  |  |  |  |  |  |  |  |
|  | 3rd year, HIV positive, 40-44 | | |  |  | 1.20 | (0.76-1.90) | |  |  |  |  |  |  |  |  |
|  | 3rd year, HIV positive, 45-49 | | |  |  | 0.34 | (0.06-2.07) | *0.146* |  |  |  |  |  |  |  |  |

**Note: The country and calendar year variables are not shown**

Table A3: Fertility rate ratios by five year age group and year before the survey from exponential regression model using all women in table A2

| Age group | Year before the survey  x  x  x  x  x  x  x | | | | | | | |
| --- | --- | --- | --- | --- | --- | --- | --- | --- |
|  | 1^st^ year  x | |  | 2^nd^ year  x | |  | 3^rd^ year  x | |
|  | FRR | 95% CI |  | FRR | 95% CI |  | FRR | 95% CI |
| 15-19 | 1.38 | (1.19-1.61) |  | 1.25 | (1.08-1.44) |  | 1.25 | (1.09-1.43) |
| 20-24 | 0.93 | (0.85-1.03) |  | 0.81 | (0.73-0.89) |  | 0.83 | (0.75-0.91) |
| 25-29 | 0.79 | (0.72-0.86) |  | 0.68 | (0.61-0.74) |  | 0.71 | (0.63-0.79) |
| 30-34 | 0.70 | (0.63-0.78) |  | 0.68 | (0.59-0.77) |  | 0.73 | (0.66-0.82) |
| 35-39 | 0.53 | (0.45-0.62) |  | 0.65 | (0.55-0.77) |  | 0.55 | (0.47-0.64) |
| 40-44 | 0.42 | (0.30-0.59) |  | 0.42 | (0.30-0.58) |  | 0.53 | (0.40-0.71) |
| 45-49 | 0.29 | (0.13-0.66) |  | 0.46 | (0.22-0.97) |  | 0.10 | (0.02-0.52) |

Figure A1: Fertility rate ratios by five year age group and year before the survey from exponential regression model using all women in table A2


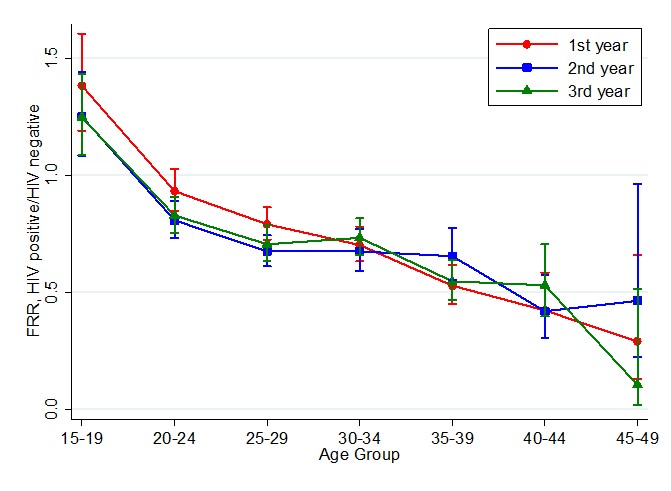


# References

**1.** ICF International. The DHS Program. *ICF International*. Available at: http://dhsprogram.com/, 2017.

**2.** Chen WJ, Walker N. Fertility of HIV-infected women: insights from Demographic and Health Surveys. *Sex Transm Infect.* Dec 2010;86 Suppl 2:ii22-27.

**3.** Marston M, Nakiyingi-Miiro J, Hosegood V, et al. Measuring the Impact of Antiretroviral Therapy Roll-Out on Population Level Fertility in Three African Countries. *PLoS One.* 2016;11(3):e0151877.

**4.** Marston M, Nakiyingi-Miiro J, Kusemererwa S, et al. The effects of HIV on fertility by infection duration: evidence from African population cohorts before ART availability: Fertility by duration of HIV infection. *AIDS.* Oct 20 2016.

**5.** Marston M, Becquet R, Zaba B, et al. Net survival of perinatally and postnatally HIV-infected children: a pooled analysis of individual data from sub-Saharan Africa. *Int J Epidemiol.* Apr 2011;40(2):385-396.

**6.** Rutstein SR, G. *Guide to DHS statistics*. Calverton, Maryland Demographic and Health Surveys, ORC Macro; 2006.
