# Supplementary material for: The relationship between HIV and fertility in the era of antiretroviral therapy in sub‐Saharan Africa: evidence from 49 Demographic and Health Surveys
Source: Trop Med Int Health. 2017 Oct 24;22(12):1542–50. doi: 10.1111/tmi.12983 (PMC5716842; doi:10.1111/tmi.12983)
Supplement: Supplementary file 2 — Supplementary Digital Content 2. [file TMI-22-1542-s002.docx]

# **Supplementary Digital Content 2:**

#

**“The relationship between HIV and fertility in the era of antiretroviral therapy in sub Saharan Africa – Evidence from 48 Demographic & Health Surveys”**

**Milly Marston, Basia Zaba, Jeffrey W. Eaton**

## Extra tables and figures

Figure A1: Urban, rural fertility rate ratio for HIV negative women (left) and HIV prevalence urban rural ratio (right) on the log scale. In all regions, HIV prevalence was higher while fertility was lower in urban areas than rural. This suggests that the lower fertility among HIV positive women may be partially confounded by urban/rural differences in fertility and HIV.

Figure A2: Age specific fertility rate ratio comparing HIV positive to HIV negative women in the year before the survey, adjusting for country and year and the interaction between years before survey and HIV status, compared to Chen and Walker estimates..

Table A1: Adjusted age specific fertility rate ratio comparing HIV positive to HIV negative women.

|  |  | Southern Africa | | | | |  | East and mid-Africa | | | | |  | West and Central Africa | | |  |  |
| --- | --- | --- | --- | --- | --- | --- | --- | --- | --- | --- | --- | --- | --- | --- | --- | --- | --- | --- |
|  |  | <20% |  | 20-49% |  | >50% |  | <20% |  | 20-49% |  | >50% |  | >20% |  | 20-49% |  | >50% |
| *Urban* | |  |  |  |  |  |  |  |  |  |  |  |  |  |  |  |  |  |
|  | 15-19 | 1.67 (1.37-2.04) |  | 1.75 (1.43-2.14) |  | 1.83 (1.49-2.25) |  | 1.49 (1.22-1.82) |  | 1.56 (1.27-1.91) |  | 1.63 (1.33-1.99) |  | 1.48 (1.21-1.80) |  | 1.54 (1.26-1.89) |  | 1.61 (1.31-1.99) |
|  | 20-24 | 1.08 (0.94-1.24) |  | 1.13 (0.97-1.31) |  | 1.18 (1.02-1.37) |  | 0.96 (0.84-1.09) |  | 1.00 (0.87-1.16) |  | 1.05 (0.92-1.20) |  | 0.95 (0.83-1.09) |  | 0.99 (0.86-1.15) |  | 1.04 (0.90-1.21) |
|  | 25-29 | 0.92 (0.81-1.05) |  | 0.96 (0.84-1.10) |  | 1.00 (0.87-1.16) |  | 0.82 (0.72-0.93) |  | 0.85 (0.75-0.98) |  | 0.89 (0.78-1.02) |  | 0.81 (0.71-0.92) |  | 0.85 (0.74-0.97) |  | 0.89 (0.77-1.03) |
|  | 30-34 | 0.70 (0.59-0.82) |  | 0.73 (0.61-0.87) |  | 0.76 (0.64-0.91) |  | 0.62 (0.53-0.73) |  | 0.65 (0.55-0.77) |  | 0.68 (0.58-0.80) |  | 0.62 (0.53-0.72) |  | 0.64 (0.54-0.76) |  | 0.67 (0.57-0.80) |
|  | 35-39 | 0.56 (0.45-0.70) |  | 0.59 (0.47-0.74) |  | 0.61 (0.49-0.77) |  | 0.50 (0.40-0.63) |  | 0.52 (0.41-0.66) |  | 0.54 (0.43-0.68) |  | 0.49 (0.39-0.62) |  | 0.52 (0.41-0.65) |  | 0.54 (0.43-0.68) |
|  | 40-44 | 0.47 (0.29-0.74) |  | 0.49 (0.30-0.78) |  | 0.51 (0.32-0.81) |  | 0.41 (0.26-0.66) |  | 0.43 (0.27-0.69) |  | 0.45 (0.28-0.72) |  | 0.41 (0.26-0.66) |  | 0.43 (0.27-0.69) |  | 0.45 (0.28-0.72) |
|  | 45-49 | 0.07 (0.01-0.66) |  | 0.07 (0.01-0.69) |  | 0.08 (0.01-0.72) |  | 0.06 (0.01-0.58) |  | 0.07 (0.01-0.61) |  | 0.07 (0.01-0.64) |  | 0.06 (0.01-0.58) |  | 0.07 (0.01-0.61) |  | 0.07 (0.01-0.63) |
| *Rural* | |  |  |  |  |  |  |  |  |  |  |  |  |  |  |  |  |  |
|  | 15-19 | 1.39 (1.17-1.65) |  | 1.45 (1.22-1.73) |  | 1.52 (1.27-1.82) |  | 1.24 (1.04-1.47) |  | 1.29 (1.08-1.54) |  | 1.35 (1.13-1.61) |  | 1.23 (1.03-1.47) |  | 1.28 (1.07-1.54) |  | 1.34 (1.11-1.62) |
|  | 20-24 | 0.98 (0.88-1.09) |  | 1.02 (0.91-1.15) |  | 1.07 (0.94-1.22) |  | 0.87 (0.78-0.97) |  | 0.91 (0.81-1.03) |  | 0.95 (0.85-1.07) |  | 0.86 (0.76-0.98) |  | 0.90 (0.80-1.03) |  | 0.95 (0.82-1.09) |
|  | 25-29 | 0.84 (0.76-0.93) |  | 0.87 (0.79-0.97) |  | 0.92 (0.81-1.03) |  | 0.74 (0.67-0.83) |  | 0.78 (0.70-0.86) |  | 0.81 (0.73-0.91) |  | 0.74 (0.65-0.84) |  | 0.77 (0.68-0.87) |  | 0.81 (0.70-0.93) |
|  | 30-34 | 0.82 (0.73-0.92) |  | 0.86 (0.76-0.97) |  | 0.90 (0.79-1.02) |  | 0.73 (0.65-0.82) |  | 0.76 (0.68-0.86) |  | 0.80 (0.71-0.90) |  | 0.72 (0.64-0.82) |  | 0.76 (0.67-0.86) |  | 0.79 (0.69-0.91) |
|  | 35-39 | 0.60 (0.50-0.71) |  | 0.63 (0.53-0.74) |  | 0.66 (0.55-0.79) |  | 0.53 (0.45-0.63) |  | 0.56 (0.47-0.66) |  | 0.58 (0.49-0.69) |  | 0.53 (0.44-0.64) |  | 0.55 (0.46-0.66) |  | 0.58 (0.48-0.70) |
|  | 40-44 | 0.50 (0.35-0.70) |  | 0.52 (0.37-0.74) |  | 0.54 (0.38-0.77) |  | 0.44 (0.31-0.63) |  | 0.46 (0.32-0.66) |  | 0.48 (0.34-0.69) |  | 0.44 (0.31-0.63) |  | 0.46 (0.32-0.65) |  | 0.48 (0.33-0.69) |
|  | 45-49 | 0.39 (0.17-0.88) |  | 0.41 (0.18-0.92) |  | 0.43 (0.19-0.96) |  | 0.35 (0.15-0.78) |  | 0.36 (0.16-0.82) |  | 0.38 (0.17-0.85) |  | 0.34 (0.15-0.78) |  | 0.36 (0.16-0.81) |  | 0.38 (0.17-0.85) |

Model: hivstatus**X**agegrp hivstatus**X**agegrp**X**residence coverage**X**hivstatus epigrp**X**hivstatus yearbefore**X**i.hivstatus**X**agegrp_fiveyr year country

Table A2: Adjusted age specific fertility rate ratio comparing HIV positive to HIV negative excluding person time prior to first sex.

| Sexually | | Southern Africa | | | | |  | East and Mid Africa | | | | |  | West and Central Africa | | |  |  |
| --- | --- | --- | --- | --- | --- | --- | --- | --- | --- | --- | --- | --- | --- | --- | --- | --- | --- | --- |
| Active | | <20% |  | 20-49% |  | >50% |  | <20% |  | 20-49% |  | >50% |  | >20% |  | 20-49% |  | >50% |
| *Urban* | |  |  |  |  |  |  |  |  |  |  |  |  |  |  |  |  |  |
|  | 15-19 | 1.00 (0.82-1.21) |  | 1.04 (0.85-1.27) |  | 1.13 (0.92-1.39) |  | 0.92 (0.76-1.11) |  | 0.96 (0.78-1.16) |  | 1.04 (0.85-1.27) |  | 0.96 (0.79-1.16) |  | 1.00 (0.83-1.21) |  | 1.09 (0.89-1.33) |
|  | 20-24 | 0.87 (0.76-1.00) |  | 0.91 (0.79-1.06) |  | 0.99 (0.86-1.15) |  | 0.80 (0.71-0.91) |  | 0.84 (0.73-0.96) |  | 0.91 (0.80-1.04) |  | 0.84 (0.74-0.96) |  | 0.88 (0.76-1.01) |  | 0.95 (0.82-1.11) |
|  | 25-29 | 0.83 (0.73-0.95) |  | 0.87 (0.76-0.99) |  | 0.94 (0.82-1.09) |  | 0.76 (0.67-0.87) |  | 0.80 (0.70-0.91) |  | 0.87 (0.76-0.99) |  | 0.80 (0.70-0.91) |  | 0.83 (0.73-0.95) |  | 0.91 (0.79-1.05) |
|  | 30-34 | 0.64 (0.55-0.76) |  | 0.67 (0.56-0.80) |  | 0.73 (0.61-0.87) |  | 0.59 (0.51-0.69) |  | 0.62 (0.52-0.73) |  | 0.67 (0.57-0.79) |  | 0.62 (0.53-0.73) |  | 0.65 (0.55-0.77) |  | 0.70 (0.60-0.83) |
|  | 35-39 | 0.52 (0.41-0.65) |  | 0.54 (0.43-0.68) |  | 0.59 (0.47-0.74) |  | 0.48 (0.38-0.60) |  | 0.50 (0.40-0.63) |  | 0.54 (0.43-0.68) |  | 0.50 (0.40-0.63) |  | 0.52 (0.41-0.66) |  | 0.57 (0.45-0.72) |
|  | 40-44 | 0.43 (0.27-0.69) |  | 0.45 (0.28-0.72) |  | 0.49 (0.31-0.79) |  | 0.40 (0.25-0.64) |  | 0.42 (0.26-0.66) |  | 0.45 (0.28-0.72) |  | 0.42 (0.26-0.67) |  | 0.44 (0.27-0.70) |  | 0.47 (0.30-0.76) |
|  | 45-49 | 0.07 (0.01-0.62) |  | 0.07 (0.01-0.64) |  | 0.08 (0.01-0.70) |  | 0.06 (0.01-0.57) |  | 0.06 (0.01-0.59) |  | 0.07 (0.01-0.64) |  | 0.06 (0.01-0.59) |  | 0.07 (0.01-0.62) |  | 0.07 (0.01-0.67) |
| *Rural* | |  |  |  |  |  |  |  |  |  |  |  |  |  |  |  |  |  |
|  | 15-19 | 0.95 (0.81-1.12) |  | 0.99 (0.83-1.17) |  | 1.07 (0.90-1.28) |  | 0.87 (0.74-1.03) |  | 0.91 (0.77-1.07) |  | 0.99 (0.83-1.17) |  | 0.91 (0.77-1.08) |  | 0.95 (0.80-1.13) |  | 1.03 (0.86-1.24) |
|  | 20-24 | 0.87 (0.78-0.97) |  | 0.90 (0.80-1.02) |  | 0.99 (0.87-1.12) |  | 0.80 (0.71-0.89) |  | 0.83 (0.74-0.94) |  | 0.91 (0.80-1.02) |  | 0.84 (0.74-0.95) |  | 0.87 (0.77-0.99) |  | 0.95 (0.82-1.09) |
|  | 25-29 | 0.79 (0.71-0.87) |  | 0.82 (0.74-0.91) |  | 0.89 (0.79-1.00) |  | 0.72 (0.65-0.80) |  | 0.75 (0.68-0.83) |  | 0.82 (0.73-0.91) |  | 0.76 (0.67-0.86) |  | 0.79 (0.70-0.89) |  | 0.86 (0.75-0.99) |
|  | 30-34 | 0.78 (0.70-0.88) |  | 0.81 (0.72-0.92) |  | 0.89 (0.78-1.01) |  | 0.72 (0.64-0.81) |  | 0.75 (0.66-0.84) |  | 0.81 (0.72-0.92) |  | 0.75 (0.66-0.86) |  | 0.78 (0.69-0.89) |  | 0.85 (0.74-0.98) |
|  | 35-39 | 0.57 (0.48-0.68) |  | 0.59 (0.50-0.70) |  | 0.65 (0.54-0.77) |  | 0.52 (0.44-0.62) |  | 0.54 (0.46-0.64) |  | 0.59 (0.50-0.71) |  | 0.55 (0.46-0.66) |  | 0.57 (0.48-0.68) |  | 0.62 (0.51-0.75) |
|  | 40-44 | 0.47 (0.33-0.67) |  | 0.49 (0.35-0.70) |  | 0.54 (0.38-0.76) |  | 0.43 (0.31-0.62) |  | 0.45 (0.32-0.64) |  | 0.49 (0.35-0.70) |  | 0.46 (0.32-0.65) |  | 0.47 (0.33-0.68) |  | 0.52 (0.36-0.74) |
|  | 45-49 | 0.37 (0.17-0.84) |  | 0.39 (0.17-0.87) |  | 0.42 (0.19-0.95) |  | 0.34 (0.15-0.77) |  | 0.36 (0.16-0.80) |  | 0.39 (0.17-0.88) |  | 0.36 (0.16-0.81) |  | 0.38 (0.17-0.85) |  | 0.41 (0.18-0.92) |

Model: hivstatus**X**agegrp hivstatus**X**agegrp**X**residence coverage**X**hivstatus epigrp**X**hivstatus yearbefore**X**i.hivstatus**X**agegrp_fiveyr year country if sexually active
